# Supplementary material for: Criterion validity of the Saltin-Grimby Physical Activity Level Scale in adolescents. The Fit Futures Study
Source: PLoS One. 2022 Sep 1;17(9):e0273480. doi: 10.1371/journal.pone.0273480 (PMC9436064; doi:10.1371/journal.pone.0273480)
Supplement: S1 Table — (DOCX) [file pone.0273480.s001.docx]

|  | **Invalid wear time** | **Valid wear time** |
| --- | --- | --- |
| **Sex  *n (%)*** | **380 (39.9)** | **572 (60.1)** |
| Girls | 148 (31.7) | 319 (68.3)* |
| Boys | 232 (47.8) | 253 (52.2) |
| **BMI category *n (%)*** | 379 (39.9) | 570 (60.1) |
| Under- and normalweight | 312 (40.6) | 457 (59.4) |
| Overweight and obese | 67 (38.5) | 113 (62.8) |
| **Study specialization *n (%)*** | **380 (39.9)** | **572 (60.1)** |
| Vocational | 224 (48.4) | 240 (58.7) |
| General | 114 (29.5) | 273 (70.5)* |
| Sports | 43 (41.3) | 61 (58.7) |
| **Parents´ education *n (%)*** | 377 (39.8) | 570 (60.2) |
| Do not know | 91 (44.6) | 113 (54.4) |
| Primary/high school | 109 (39.5) | 167 (60.5) |
| University <4 years | 63 (35.4) | 115 (64.6) |
| University ≥4 years | 114 (39.4) | 175 (60.6) |
| **Self-reported health *n (%)*** | **379 (40.0)** | **569 (60.0)** |
| Very poor/poor | 21 (41.2) | 30 (58.8) |
| Neither good nor poor | 87 (42.2) | 119 (57.8) |
| Good | 184 (40.0) | 276 (60.0) |
| Excellent | 87 (37.7) | 144 (62.3) |

**Supplementary Table 1.** Distribution of valid and invalid accelerometry wear time.

BMI=body mass index. There are slightly different N within strata due to missing confounders in some individuals. *Significant higher proportion valid wear time, p<0.001.
