# Supplementary material for: Criterion validity of the Saltin-Grimby Physical Activity Level Scale in adolescents. The Fit Futures Study
Source: PLoS One. 2022 Sep 1;17(9):e0273480. doi: 10.1371/journal.pone.0273480 (PMC9436064; doi:10.1371/journal.pone.0273480)
Supplement: S2 Table — The Fit Futures Study 2010–2011. (DOCX) [file pone.0273480.s002.docx]

**Supplementary Table 1.**
*Accelerometry wear time by BMI, study specialization, parental education and self-perceived health.* *The Fit Futures Study 2010-2011*

| **Wear time (h)** |  |
| --- | --- |
| **Sex** | **Mean ± SD** |
| Girls | 14.00 ± 1.10 |
| Boys | 14.25 ± 1.22 |
| **BMI** **category** | **Mean ± SD** |
| Underweight or normal weight | 14.11 ± 1.16 |
| Overweight or obese | 14.14 ± 1.09 |
| **Study specialization** | **Mean ± SD** |
| Vocational | 14.19 ± 1.10 |
| General | 14.13 ± 1.18 |
| Sports | 14.13 ± 1.18 |
| **Parents' education** | **Mean ± SD** |
| Do not know | 14.17 ± 1.10 |
| Primary/high school | 14.16 ± 1.16 |
| University <4 years | 14.21 ± 1.10 |
| University ≥4 years | 13.97 ± 1.19 |
| **Self-perceived health** | **Mean ± SD** |
| Very poor/poor | 13.94 ± 1.07 |
| Neither good nor poor | 14.02 ± 1.23 |
| Good | 14.19 ± 1.18 |
| Excellent | 14.06 ± 1.00 |

BMI=body mass index, SD=standard deviation.
