# Supplementary material for: Criterion validity of the Saltin-Grimby Physical Activity Level Scale in adolescents. The Fit Futures Study
Source: PLoS One. 2022 Sep 1;17(9):e0273480. doi: 10.1371/journal.pone.0273480 (PMC9436064; doi:10.1371/journal.pone.0273480)

PONE-D-21-01038
Criterion validity of the Saltin…

Intro:

Thank you for taking the time to put together this manuscript. Your efforts should be applauded. I hope my comments are helpful. They are suggestions. If you feel differently, or that I made a mistake, please support your point for the editor and future reviewers.

General Comments

I enjoyed your paper. The literature needs good research building up the validity evidence for physical activity measures in the populations where they are used, with the outcomes that are important, and the health-related modifiers that are often overlooked.

- With validity and reliability, we are generally building evidence that supports the use of a measure for a set of people, in a given situations, with reference to specific outcomes from the measure. My preference is that we talk about "building evidence for the validity of XX" and not "we are validating XX". I understand that this is more of an individual preference, but single studies with specific groups and only 1 outcome are often used to support using measures in other groups with a different outcome from that measure. Part of this is because we often talk in our results and discussions about "validating XX", which implies the instrument is good, not that it has support for use in similar contexts.

- For the models in table 3 and table 4 (correlation and regression) we need some indication of the distributions of the 4 SGPAL categories in those groups. Similar to how you show us girls vs boys. You could do this with an expansion of table 2. Just add 4 columns to right with each SGPAL category. The reason we want to know this is to understand the differences better. If the Overweight BMI category has 90% of people in categories 1 and 2 then the differences, we see in Table 4 (8.97 vs 5.33) per category need to be interpreted in this context.

- You might need to look at all the scatter plots and visually inspect for outliers driving correlation up or down. If you did this, mention it in the methods.

- Not needed but you might consider adding the relationship between SGPAL and a "meeting recommendation" outcome computed with Accelerometer MVPA or steps. You could show % in each SGPAL group getting more or less than 60 minutes of MVPA per day. Just more support for using SGPAL in the context of public health recommendations.

- Also not need but you could add to results (and evidence) by looking at accelerometer and SGPLAY score differences by group (sex, BMI, edu…). To see if SGPLAY identifies the same difference as accelerometer outcomes.
    - Example: Do we see the same difference in MVPA and SGPLAY score between girls and boys?
        - Significance and percent difference
        - Maybe it would look like this

    - Boys have 43 minutes of MVPA and average SGPLAY score of 3.1
    - Girls have 37 minutes of MVPA and average SGPLAY score of 2.8
    - Boys have 16% higher MVPA minutes and 11% higher SGplay scores both are statistically significant.

- Supplement table 4 with figures to show group differences. This would be 15 plots in a supplement so we can see the change with each SGPAL level by group. Like figures I added below, but you would have each group (girls/boys) plotted, with different figures for each grouping (sex, BMI, education) and PA outcome (steps, CPM, MVPA)

- You did some very nice work with the evidence and results for different groups (Sex, BMI, Health), but the discussion of this (line 229 to 233) is lacking. There is literature in adults and children about how some demographic factors may impact validity and reliability evidence for a given questionnaire. Dig in a little more in this area and really contribute to this area of research.

Specific Comments:

Line number	Priority	Comment
39-40	moderate	Correlations of 0.40 and 0.35 are fairly close for data like this. Consider removing “moderately” and “weakly” to describe magnitude. This will apply through the manuscript (discussion also)
70-74	Low	I would prefer talking about the strength of the validity evidence rather than “is thoroughly validated”. As an example… your aim is to build validity evidence for using the SGPALS in Norwegian adolescents.
94	High	Did excluded participants (n=427) differ from included (n=572)?<br>Demographics and SGPALS ratings<br>You mention this in the discussion (line 241-247), but this information should be in the methods.
116	Low	May need another few words or sentence with more info about “study program”. What does this mean for high schoolers in Norway?
150	High	Either in methods or start of results/table 2 it would be good to report average wear times (hours per day and Days per week)<br>You should also confirm that none of the comparison groups (Sex, BMI, others in table 3) have differences in wear hours per day. Wear time at the day level is likely correlated with minutes of MVPA and Steps at ~0.25-0.40 level. If wear time is different this would impact the values, you are using to assess validity evidence in this group.
Table2 or 175	Moderate	Need to have % participants at each SGPAL level. Numbers are in table 5, but should likely be presented at start of results.<br><br>Same for Mean (SD) for all PA outcomes (MVPA, CPM, Steps)
Table 2/175	Moderate	Because a lot of your evidence is built around difference for various demographic variables, it would be beneficial to comment on how much those groups overlap. For example, Do BMI categories and Parent education overlap. Participants in lower education group tend to have higher BMI values

<table>
<tr><td>178</td><td>Low</td><td>I realize you have a range for describing the strength of the correlations, but 0.35 and 0.40 are very close in this context. Jumping from “weak” to “moderate” is over statement.</td></tr>
<tr><td>Table3</td><td>Low</td><td>Rather than “**”for almost all correlations with indicators for p-value, it would be better for your story to only highlight the differences that are important (what you focus on in text). For example

<table><tr><td>BMI</td><td>CPM</td><td>Steps</td><td>MVPA</td></tr><tr><td>Under</td><td>0.43</td><td>0.35</td><td>0.38</td></tr><tr><td>Over</td><td>0.27</td><td>0.32*</td><td>0.20*</td></tr></table>
You could pick two indicators. One for group difference for same PA outcome (bold) and one for difference across PA outcome within a group (*)</td></tr>
<tr><td>187/table 4</td><td>Moderate</td><td>Is this information from a regression model, different than the ANOVA model for results reported in table 5? May need to explain this model in stats section.</td></tr>
<tr><td>Table 4</td><td>Moderate</td><td>What about intercepts, this tells us if the rate of change differed, but not the average starting value. This would be important for group differences</td></tr>
<tr><td>Table 4</td><td>High</td><td>My guess is they are not all linear. I think you need to provide the 15 plots in a supplement so we can see the change with each SGPAL level by group. Like figure below, but you would have each group (girls/boys) plotted, with different figures for each grouping (sex, BMI, education) and PA outcome (steps, CPM, MVPA)</td></tr>
<tr><td>188/189</td><td>Moderate</td><td>This model may only be linear for steps (see plots below). For MVPA there is definitely a bigger jump moving from grp3 to grp4</td></tr>
<tr><td>Table5</td><td>Low</td><td>I like this, but I think three box plots (one for each PA outcome) would have more impact for the reader when emphasizing the differences in activity across the 4 SGPAL ratings.</td></tr>
<tr><td>207</td><td>Low</td><td>Similar to rest of literature comparing SR to Objective measures</td></tr>
<tr><td>215</td><td>Low</td><td>Not sure you mean bias</td></tr>
<tr><td>216</td><td></td><td>While it may not be a very accurate estimate of volume, table 5 suggests that you might get pretty good estimates of volume, especially if you also accounted for sex, BMI, edu, and rating of health.
You could check the precision of these estimates by looking at the measured accelerometer outcomes versus those predicted from the regression model. This would give you average errors and a way to quantify bias and average prediction error. For example,
MVPA from SGPALS rating vs MVPa from accelerometer
MVPA from SGPALS rating and Demographics vs MVPA from accelerometer</td></tr>
<tr><td>221/222</td><td></td><td>This point is important, but would be more impactful is you gave us some of the values from the adult data (26,28) for comparison. Example… If in adults the difference in steps across the 4 groups is similar (1000 steps) this would be nice to see. If adults show a similar pattern but magnitude is different (15 min of MVPA vs 8) this would also be interesting and important.</td></tr>
<tr><td>232</td><td></td><td>Why? Do we see this in what you present in tables</td></tr>
</table>

249 - 256		I don't think you need this. No one will need you to justify using acceleometers as gold standard. You also used three estimates from accelerometer which quantify different but overlapping parts of the PA puzzle. This is strength!
263		Would be better here, or in methods, for you to present the ICC for your data. ICC for all three outcomes over all days measured.
265/266		Did you look at the levels over days (1 vs 2,3,4,5,6,7) ?<br>You could look at this an see if you think there was reactivity.
274-275		I think you need to provide the error levels and bias in this prediction at the individual and group levels before saying that volume and intensity should not be estimated.

Data from table 5.
Steps seems linear…

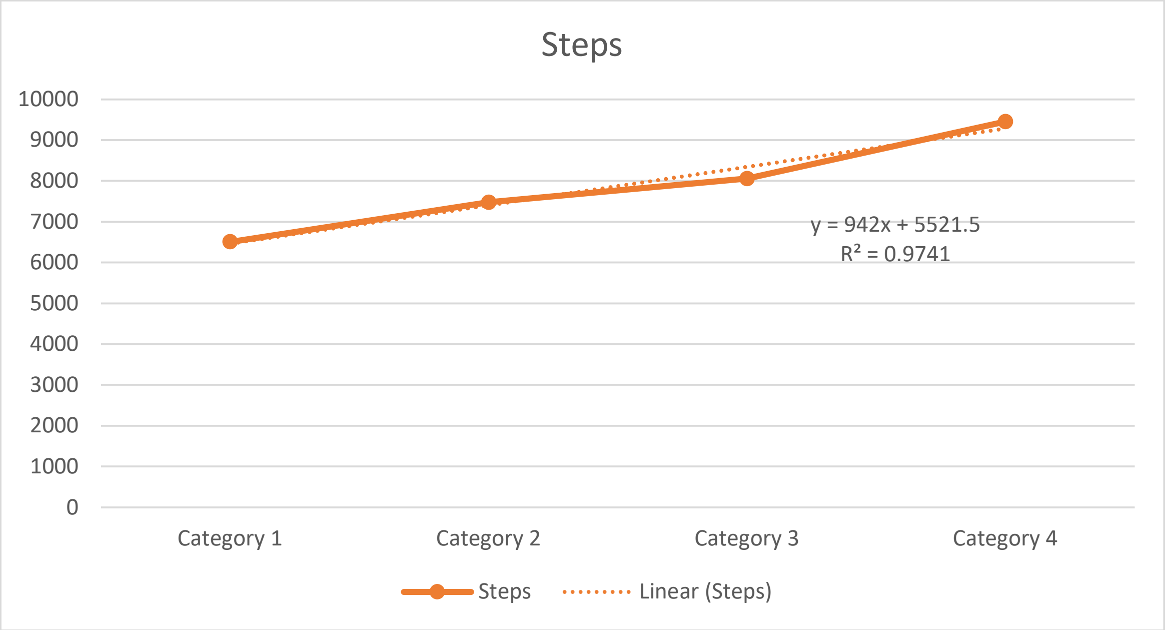

CPM maybe not linear.

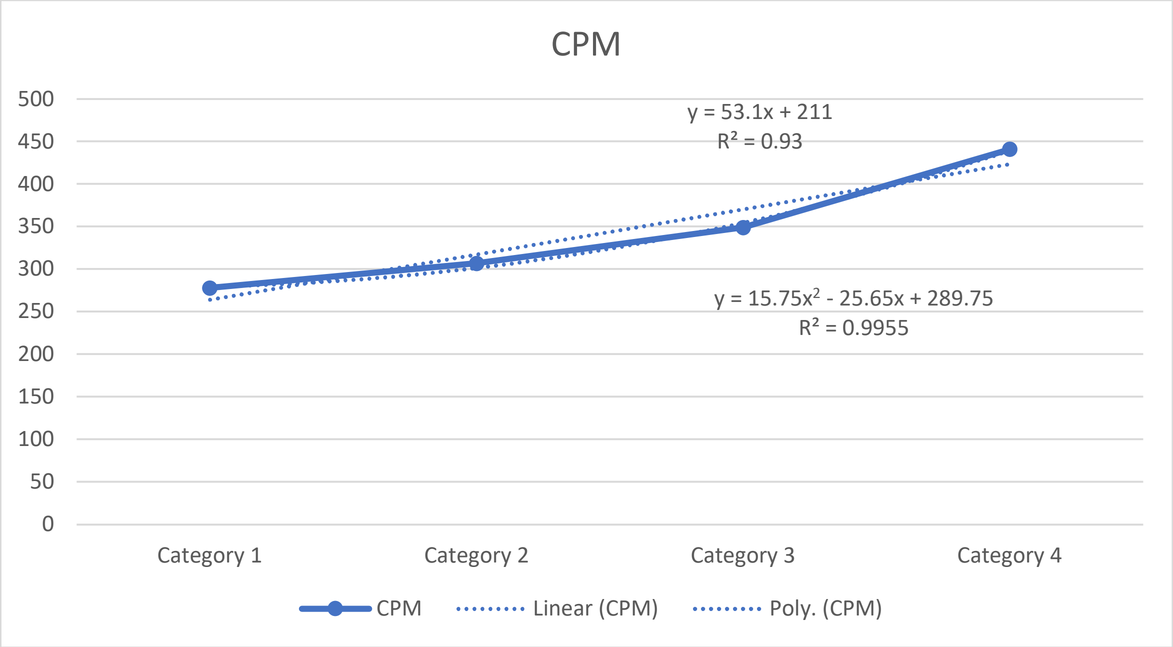

MVPA linear grp 1,2,3 then jump for grp 4.

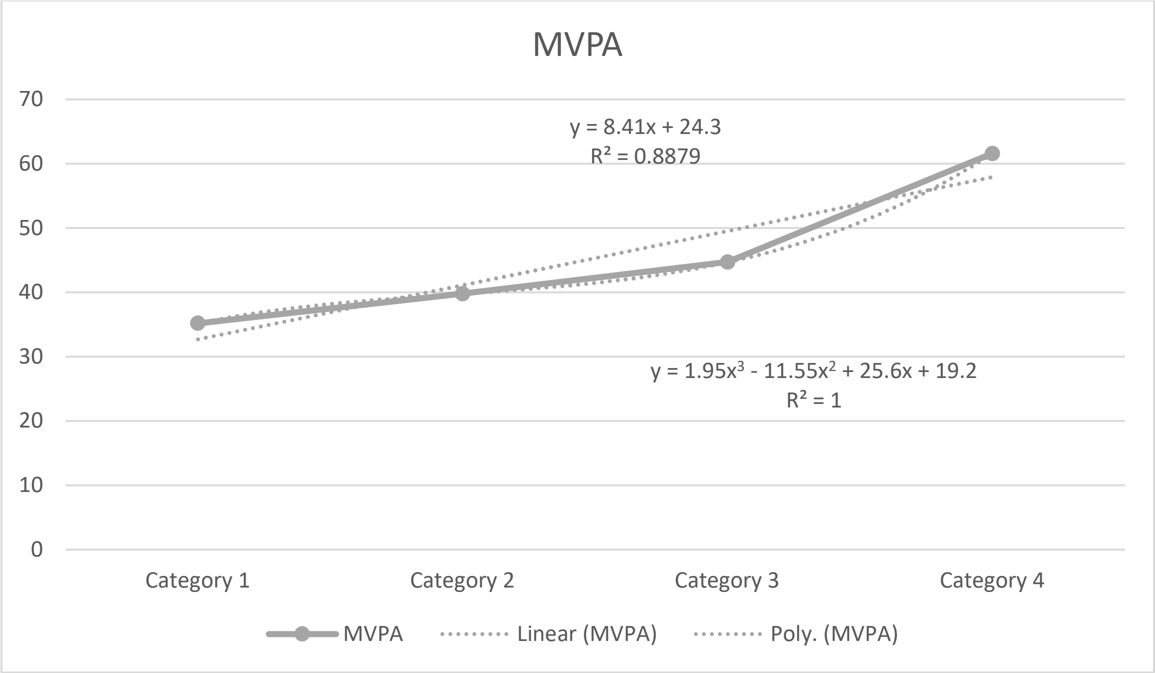

Supplement: Attachment — Submitted filename: Review_PONE-D-21-01038.pdf [file pone.0273480.s003.pdf]
