# Supplementary material for: Criterion validity of the Saltin-Grimby Physical Activity Level Scale in adolescents. The Fit Futures Study
Source: PLoS One. 2022 Sep 1;17(9):e0273480. doi: 10.1371/journal.pone.0273480 (PMC9436064; doi:10.1371/journal.pone.0273480)
Supplement: Attachment — Submitted filename: Response to reviewer.docx [file pone.0273480.s005.docx]

# Response to reviewers

All line-references are to the line number in the revised tracked changes document.

1. Thank you for the useful suggestions, we have expanded the discussion on page 15-17 regarding comparison with other validation papers and indicated that the weak correlations between self-report and objective PA measures found here are not unique, and the value of our findings.

2. We have added some sentences on future research on page 19, based on your valuable suggestions.
